# Does aging amplify the rule-based efficiency effect in action selection?

**DOI:** 10.3389/fpsyg.2023.1012586

**Published:** 2023-03-01

**Authors:** Jean P. P. Scheib, Sarah E. M. Stoll, Jennifer Randerath

**Affiliations:** ^1^Department of Psychology, University of Konstanz, Konstanz, Germany; ^2^Lurija Institute for Rehabilitation Science and Health Research, Kliniken Schmieder, Allensbach, Germany; ^3^Outpatient Unit for Research, Teaching and Practice, Faculty of Psychology, University of Vienna, Vienna, Austria

**Keywords:** action selection, action planning, motor cognition, end-state comfort, implementation intentions, drift diffusion

## Abstract

When it comes to the selection of adequate movements, people may apply varying strategies. Explicit if-then rules, compared to implicit prospective action planning, can facilitate action selection in young healthy adults. But aging alters cognitive processes. It is unknown whether older adults may similarly, profit from a rule-based approach to action selection. To investigate the potential effects of aging, the Rule/Plan Motor Cognition (RPMC) paradigm was applied to three different age groups between 31 and 90 years of age. Participants selected grips either instructed by a rule or by prospective planning. As a function of age, we found a general increase in a strategy-specific advantage as quantified by the difference in reaction time between plan- and rule-based action selection. However, in older age groups, these differences went in both directions: some participants initiated rule-based action selection faster, while for others, plan-based action selection seemed more efficient. The decomposition of reaction times into speed of the decision process, action encoding, and response caution components suggests that rule-based action selection may reduce action encoding demands in all age groups. There appears a tendency for the younger and middle age groups to have a speed advantage in the rule task when it comes to information accumulation for action selection. Thus, one influential factor determining the robustness of the rule-based efficiency effect across the lifespan may be presented by the reduced speed of information uptake. Future studies need to further specify the role of these parameters for efficient action selection.

## 1. Introduction

The ability to interact with objects is an essential aspect of everyday life. For example, appropriate initial grasping facilitates object manipulation by guaranteeing a comfortable movement end-state (Rosenbaum et al., 2012). For instance, when opening a cabinet door, the handle can be manipulated with either a pronated or supinated grip. The choice of grip depends on the handle’s location (above or below the actor) and the subsequent movement (e.g., the door opens downward or upward). A vast range of studies has demonstrated that many actions are selected by prospective planning (Rosenbaum et al., 2012; Ansuini et al., 2016; Scharoun et al., 2016a; Scharoun Benson et al., 2018). However, object-related actions can be diminished after brain damage (Stoll et al., 2022a). Difficulties can begin as early as the planning stages of the object-related grasping movements. While healthy individuals easily select the appropriate grip in anticipation of the movement’s comfortable end-state and efficiently open the door, patients with impairments in prospective planning may struggle significantly to solve these basic actions (Hermsdorfer et al., 1999; Buxbaum et al., 2005; Randerath et al., 2009).

Furthermore, studies investigating the effects of healthy aging have reported that even healthy older adults tend to show fewer end-state comfortable grips than young adults (Scharoun et al., 2016b; Stöckel et al., 2017; Wunsch et al., 2017). This effect seems to be enhanced by increased task complexity (Wang et al., 2020). Reduced functional and structural integrity in the brain, especially in frontal and parietal regions and their connections with other cortical and subcortical areas, may drive the frequently observed finding of diminished executive functions (including working memory), processing speed (Garcia-Cabello et al., 2021), and motor cognitive decline (Heuninckx et al., 2005; Goldenkoff et al., 2021). It has been discussed that older adults may adopt more adaptive strategies to better compensate for age-related changes in motor planning (Wang et al., 2020) to sustain levels of cognitive function (Dockree et al., 2015; Samu et al., 2017). Those compensatory mechanisms include, for example, higher activation in frontoparietal areas typically associated with motor cognitive behavior.

Indeed, there could be entirely alternative approaches to action selection besides prospective planning that could lead to the production of the very same actions. For example, implicit and explicit rules support behaviors such as hitting the brake at a red traffic light (Bunge, 2004). Such rule-based approaches contribute to action selection as fixed stimulus-response mappings in contrast to the aforementioned plan-based movement selection, which involves flexible stimulus-response mappings. If-then rules (in some contexts referred to as Implementation Intentions) have been shown to facilitate many cognitive tasks (Gollwitzer and Sheeran, 2006; Wieber et al., 2015). Several motor cognitive studies have demonstrated that using such rules leads to shorter action initiation latency and reduced error rates compared to using a prospective planning approach when producing the same actions (Randerath et al., 2013, 2015, 2017; Scheib et al., 2018; Stoll et al., 2022b). It has been hypothesized that rule-based action selection may have the potential to be particularly effective in improving the successful selection of grips in persons with difficulties in prospective planning. Thus far, the latter studies have only implemented the “Rule/Plan Motor Cognition paradigm” (RPMC) in young, healthy adults. Here, we for the first time, study the differential effects of plan- and rule-based action selection across the adult lifespan.

**FIGURE 1:**
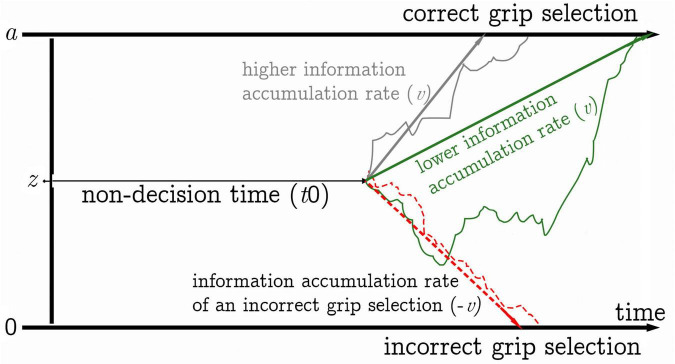
Graphical representation of the drift diffusion model (DDM).

To further unravel age-affected components subserving action selection via rules or planning, we additionally applied drift diffusion modeling (DDM) (Ratcliff, 1978; Ratcliff and McKoon, 2008) to reaction time (RT) data gathered with the RPMC paradigm in the current study. Diffusion modeling has been used in many fields of psychology including research on memory and perception research (Ratcliff et al., 2001, 2004a,b,^2006^, ^2007^; Thapar et al., 2003; Spaniol et al., 2006) to decompose reaction time distributions into cognitively meaningful components. Drift diffusion parameters may enable a deeper understanding of rule- and plan-based movement-selection approaches (Scheib et al., 2018; see Figure 1). In binary decision tasks (e.g., selection of pronated vs. supinated grips), the diffusion model allows for the statistical decomposition of reaction time distributions into cognitively meaningful parameters reflecting (among others) the speed of the decision process as the rate of information accumulation (drift rate, *v*), response caution as the distance between decision thresholds (boundary separation, *a*), and the duration of non-decision components (e.g., stimulus encoding, preparation of motor response, task switching, visualization) combined in the non-decision time parameter *t*_0_ (Voss et al., 2004). In the DDM, decision processes are modeled as noisy random processes originating from a starting point located between an upper decision boundary *a* and a lower decision boundary 0. A decision is made when the decision process reaches one of the decision boundaries. A lower value of the *a* parameter (which represents response caution), indicates that less information is required to come to a decision (i.e., a low value of *a* implies that the distance between decision boundaries is low), leading to faster reaction times and a higher error probability (more liberal response criterion). A lower value of the speed of the decision process parameter *v* points toward less efficient processing of decision related information. Differences in *t*_0_ between approaches to action selection show that processes not directly involved in the decision differ. In our previous study with healthy young adults, we found significantly higher drift rates (*v* parameter) in the rule-task than in the plan-task, suggesting, that decision-relevant information processing can be more efficient in the rule-task than in the plan-task (Scheib et al., 2018). The DDM is of particular value in the context of aging, as diffusion parameters have previously been shown to be sensitive to aging in a variety of cognitive tasks such as lexical-decision tasks (Ratcliff et al., 2004b), signal detection tasks (Ratcliff et al., 2001), letter discrimination tasks (Thapar et al., 2003), memory (Spaniol et al., 2006), and rapid two-choice decisions in general (Ratcliff et al., 2006, 2007). The most stable results in the context of aging have been an increase in non-decision times (Dully et al., 2018). However, the effects of aging on drift rate appear to depend more on the experimental task (Dully et al., 2018). A recent meta-analysis on the effects of aging on DDM parameters (Theisen et al., 2021) found that older adults exhibit lower drift rates in perceptual and memory tasks but increased drift rates in lexical decision tasks.

In the current study, we attempt to determine the effects of aging firstly on task related efficiency effects reflected by response times and secondly, the effects of aging on DDM parameters in the context of a motor cognitive task using the RPMC paradigm.

### 1.1. Hypothesis

In our current study, participants between 31 and 90 years of age had to select a grip and subsequently rotate a handle on a rotation device. Participants were asked to solve the task either instructed by a rule (rule-task) or by utilizing the most comfortable grip, which was prospectively planned in accordance with *end-state comfort* (plan-task).

Previous studies have suggested that older adults show lower performance levels in action planning tasks (Scharoun et al., 2016b; Stöckel et al., 2017; Wunsch et al., 2017). We hypothesized an increase in the advantage of rule-based action selection relative to plan-based action selection in older age. Thus, participants of older age should demonstrate an amplified rule-based efficiency effect. We quantified increased efficiency in terms of lower RTs and higher drift rates (*v*).

As will be discussed, our results suggest a more nuanced picture than the one proposed.

## 2. Methods

The experiment was approved by the ethics committee of the University of Konstanz (statement no. 10/2014). All participants gave written informed consent. The study was conducted in accordance with the Declaration of Helsinki.

### 2.1. Participants

We recruited a community sample of *N* = 81 participants between 31 and 90 years old with normal or corrected-to-normal vision. We excluded *n* = 1 participants with lateralization quotients <60 (Salmaso and Longoni, 1985) to include only participants with a rather strong preference for the right hand in order to reduce the potential influence of a confounding factor, because differences between right- and left-handed subjects have been reported for functional brain organization and related behavior (Goldenberg, 2013; Scharoun et al., 2016a; Kroliczak et al., 2021). After screening for cognitive impairment and early signs of dementia with DemTect (Kessler et al., 2000, 2014), we excluded another *n* = 2 participants. To achieve equal sample sizes per age group (young = 31–50 years, middle = 51–70 years, old = 71–90 years) while keeping the distribution of ages as uniform as possible, we randomly excluded six more participants. The final sample included 72 participants. About half of each age group performed the task with their dominant right hand and the others with their non-dominant left hand. Participants received 20 EUR for their participation. Instructions were given in German. The experimenter confirmed language fluency.

### 2.2. Materials and procedure

**FIGURE 2:**
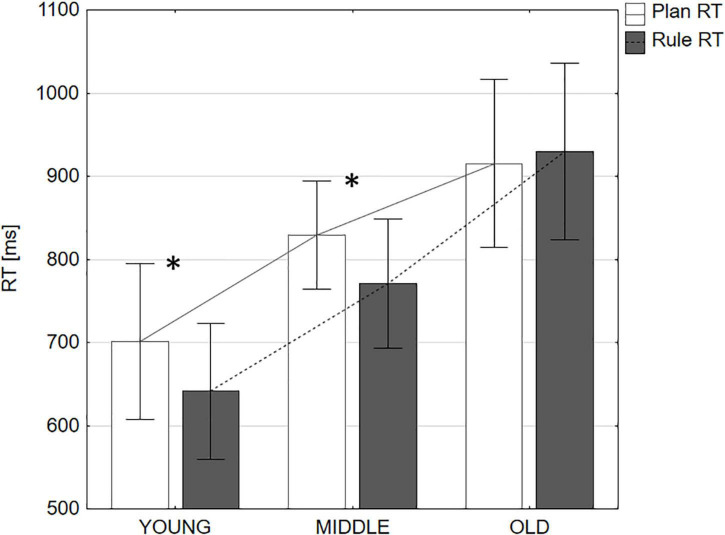
Plan- and rule-task mean reaction times (RT) per age group are given in milliseconds (ms). Error bars represent 95% confidence intervals. Significant median differences within age groups are marked with *.

Each participant was tested in a single 60 min session. The stimuli were presented with SuperLab 5 (Cedrus Corporation, San Pedro, CA, USA) on a 24-inch monitor. The monitor, rotation apparatus, and response pad were placed on a table. Table height was adjusted to center the monitor’s viewable area at each participant’s eye level. Participants wore Translucent Technologies PLATO visual occlusion goggles,^1^ which can switch between opaque (shut) and clear (open) lens states. They were used to control the visibility of the monitor and the RPMC-apparatus. The purpose-built RPMC-apparatus consisted of a rotatable handle mounted on a stand centered in front of and level with the center of the monitor. Two light-emitting diodes were mounted on the handle (see “section 2 Methods” and Figure 2 in related article).^2^ Before each trial, participants heard an acoustic prompt (1,000 ms duration). The goggles opened after a randomized inter-stimulus interval of 500, 800, or 1,100 ms. Participants released the response button, reached for and grasped the handle, and subsequently rotated the handle. The apparatus blocked further rotation when the handle was rotated far enough. They then returned the hand to the response button, which triggered closing of the goggles. The experiment consisted of 64 trials presented in blocks of 32 plan trials or 32 rule trials, respectively. Half of the trials in each block required an underhand (supinated) grip to rotate the handle; the other half had to be solved with an overhand (pronated) grip. Trials were presented in a pseudo-randomized order, so there were no more than three consecutive trials with the same grip. Grip requirements were achieved by varying on-screen rotation targets and light placement on the handle (Scheib et al., 2018).

The handle had a differently colored light-emitting diode on each end. Participants were instructed to rotate the handle to align one end with a colored arrow. Which end had to be aligned was defined by the arrow’s color: the end with the same colored light (matching the arrow) had to be aligned with the arrow. Plan and rule tasks had different light color combinations (plan: green/yellow, rule: blue/magenta for half of the participants in each age group and vice versa for the other half). Note that lights on the handle were set such that applying the rule (i.e., placing the thumb on the side of the handle with the same color as the arrow) to plan trials would lead to incorrect initial grips (i.e., grips leading to uncomfortable post-rotation hand positions).

In the plan-task, participants were asked to execute the movement as comfortably as possible. They were then given an instruction card stating, “I will execute the movement as comfortably as possible.” They were asked to read the statement out loud.

For the rule-task, participants were asked to grasp the handle such that their thumb would be on the same side of the handle as the light of the same color as the arrow stimulus. Participants were shown another instruction card, which they also read out loud. It stated: “If the arrow is green, then I will place my thumb on the green side of the handle” and “If the arrow is yellow, then I will place my thumb on the yellow side of the handle.”

About half of each age group performed the task with their dominant right hand and the others with their non-dominant left hand (in the old age group 13 participants used their dominant right hand, and 11 participants used their non-dominant left hand; see Supplementary Figure 2). (Please note: originally it was planned to subsequently implement this procedure in patients who may not be able to use their dominant hand. We were not interested in analyzing hand dominance as a factor in the current study. However, Supplementary Figure 4 includes descriptive and inferential statistics including the factor hand). In both tasks, participants were asked to read and memorize the instructions presented on the card. The procedure was demonstrated, and the instructions were repeated once more before a series of four practice trials was presented before the plan and rule blocks. *N* = 35 participants started with the rule block; *n* = 36 began with the plan block (see Supplementary Figure 2).

### 2.3. Data analysis

#### 2.3.1. RT analysis

Reaction times (interval between goggles opening and button release) served as the dependent variable. Trials with grips leading to an uncomfortable end position were excluded from the analysis. In addition, trials with technical errors, premature button releases, or handle rotations in the wrong direction were also excluded. RT outliers, as identified by the Extreme Studentized Deviate test (Rosner, 1983), were removed.

Reaction time data were tested for normality using the Kolmogorov-Smirnov test, which indicated that plan- and rule-RTs in the young age group were not normally distributed (*p* ≤ 0.024). Hence, for the following analyses, non-parametric options were considered.

We compared RTs in plan- versus rule-based tasks with one Wilcoxon signed-rank test per age group (young: 31 to 50, middle: 51 to 70, and old: 71 to 90 years of age).

Rule efficiency was defined as the difference in RTs between plan- and rule-tasks. It was calculated per subject by subtracting the mean RT of the rule-task from the mean RT of the plan-task (i.e., positive values indicate greater efficiency in the rule-task and negative values indicate greater efficiency in the plan-task). Additionally, we calculated absolute values of this difference (i.e., the unsigned magnitude of the difference between plan and rule-task RTs) to receive an absolute measure of the efficiency effect. This measure reflected whether a participant performed more efficiently in either task, with 0 indicating equal efficiency.

**TABLE 1:** Group statistics for reaction times (RTs) and drift diffusion model (DDM) parameters.

	Young group	Middle group	Old group
**Reaction time (RT)**			
**Plan-task**			
*Mdn* (ms)	668.70	836.81	940.75
*M* (ms)	701.00	828.88	914.99
*SD* (ms)	221.71	153.94	239.52
**Rule-task**			
*Mdn* (ms)	625.87	741.88	895.62
*M* (ms)	641.76	770.99	929.92
*SD* (ms)	193.62	183.42	251.31
**Wilcoxon signed-rank test (plan vs. rule)**			
*T*	62.0	81.0	140.0
*p*	**0**.**012**	**0**.**049**	0.775
**DDM-parameters**			
**Boundary separation (*a*)**			
**Plan-task**			
*M*	1.99	1.63	1.85
*SD*	0.20	0.20	0.19
**Rule-task**			
*M*	1.98	2.04	2.20
*SD*	0.08	0.15	0.20
***t*-test (plan vs. rule)**			
*t*	0.18	-7.94	-6.35
*df*	46	46	46
*p*_bf_	>1	**<0**.**001**	**<0**.**001**
**Drift rate (*v*)**			
**Plan-task**			
*M*	3.42	2.91	2.44
*SD*	0.28	0.17	0.23
**Rule-task**			
*M*	3.57	3.02	2.52
*SD*	0.20	0.17	0.18
***t*-test (plan vs. rule)**			
*t*	-2.15	-2.25	-1.32
*df*	46	46	46
*p*_bf_	0.11	0.088	0.576
**Non-decision time (*t*_0_)**			
**Plan-task**			
*M* (s)	0.37	0.55	0.54
*SD*	0.02	0.02	0.04
**Rule-task**			
*M* (s)	0.34	0.42	0.50
*SD*	0.01	0.03	0.03
***t*-test (plan vs. rule)**			
*t*	6.39	17.63	5.66
*df*	46	46	46
*p*_bf_	**<0**.**001**	**<0**.**001**	**<0**.**001**

Table shows descriptive statistics for reaction times (RT) and drift diffusion model (DDM) parameters per task and age group, as well as inferential statistics showing within-subjects comparison of RT and DDM parameters for plan- versus rule-tasks (median, *Mdn*; mean, *M*; *SD*, standard deviation; s, seconds; ms, milliseconds; *p*_bf_, Bonferroni corrected *p*-value). Bold font denotes *p*-values < 0.05.

Since neither the rule- nor the absolute values of the efficiency effect were normally distributed, we applied Kendall’s tau to analyze the correlation of age with rule efficiency on the one hand as well as the absolute efficiency effect on the other hand.

#### 2.3.2. Post-hoc analysis

To test distribution differences between the three age groups, we calculated three Brown-Forsythe tests for variance homogeneity centered around the group medians and corrected for multiple comparisons with the Bonferroni procedure.

#### 2.3.3. DDM analysis

We analyzed the DDM parameters drift rate (*v*: the rate of information accumulation; speed of the decision process), boundary separation (*a*: the distance between decision thresholds; response caution), and the duration of non-decision components (*t*_0_: which is thought to reflect processes such as stimulus encoding, preparation of motor response, visualization; action encoding). Lower values of the *v*-parameter would indicate less efficient processing of decision related information. Lower values in *a* would indicate a more liberal decision process going along with a higher probability for producing errors. Higher values in the *t*_0_ parameter would reflect heightened processing of information not directly involved in the decision.

For DDM analysis with *fast-dm-30.2* (Voss and Voss, 2007), we pooled raw reaction times (including trials containing grip errors) of all subjects belonging to a respective age group into “super subject” datasets (Voss et al., 2013), resulting in a young, middle, and old “super datasets.” This pooling of data was necessary due to the low number of trials per individual participant.

We then used boxplots to identify and remove outlier trials from log-transformed data (Voss et al., 2015). This was done separately for trials that resulted in incorrect and correct grip selection.

Next, we estimated DDM parameters for each super subject (with parameters *v*, *t*_0_, *a*, inter-trial-variability of *v*, inter-trial-variability of *t*_0_, and differences in speed of response execution estimated dependent on task, and relative starting point set to 0.5, i.e., bias-free). We then used those estimates (and settings) to create 24 simulated plan and rule RT datasets per age group, containing 4,096 trials each, with the *fast-dm* construct-samples tool to approximate the magnitude of effects.

We recovered DDM parameters from the simulated datasets and calculated a series of *t*-tests for drift rate (*v*), non-decision time (*t*_0_), and decision boundary separation (*a*) for each age group, comparing rule and plan-tasks. We corrected for multiple comparisons using the Bonferroni method.

For further tables, figures, descriptive and post hoc analyses suggested by the reviewers see Supplementary Figures 1–6 and Supplementary Tables 1–4.

## 3. Results

### 3.1. RTs

Descriptive statistics for RTs and results are summarized in Table 1.

We compared tasks within groups using the Wilcoxon test to analyze the effect of aging on rule versus plan performance, see Figure 1. In the young and middle age groups, rule RTs were significantly faster than plan RTs. However, rule RTs and plan RTs were not significantly different in the old age group.

Thus, on the group level, a relatively higher efficiency of the rule-task could not be demonstrated in the old age group. Accordingly, there was no significant linear correlation between age and higher rule efficiency, *r*_τ_ = −0.05, *p* = 0.505. However, the absolute magnitude of the efficiency effect correlated significantly with age, *r*_τ_ = 0.28, *p* = 0.001, indicating an increase in task divergence between the plan and the rule task with age.

### 3.2. *Post-hoc* tests

**FIGURE 3:**
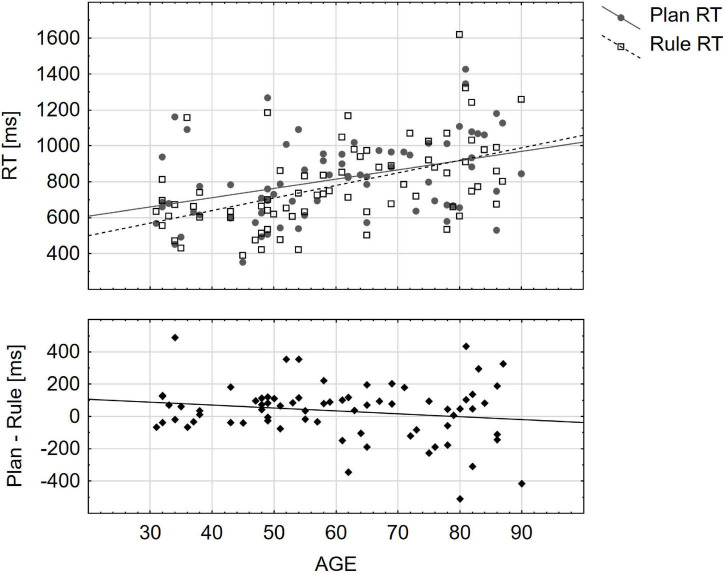
The figure depicts task-specific **(upper panel)** and rule efficiency **(lower panel)** reaction time (RTs) distributions, demonstrating a change in strategy advantages with increasing age. While young participants appeared quicker in rule-based action selection, older adults varied more strongly in which approach was more advantageous. **(Upper panel)** Mean plan- and rule-task RT for each participant, plotted against each participant’s age in years. **(Lower panel)** Rule efficiency effect (mean rule-task RT subtracted from mean plan-task RT) for each participant plotted against each participant’s age. See Supplementary Figure 2 for a breakdown of the **(lower panel)** by hand and first task.

We observed that the spread of task RT differences (calculated as Plan RT–Rule RT, see Figure 3) was widest in the old age group (*SD* = 228.50 ms, *Min* = −510.42 ms, *Max* = 434.47 ms) and narrowest in the young age group (*SD* = 115.79 ms, *Min* = −65.73 ms, *Max* = 488.36 ms). This prompted the question, whether the three age groups significantly differed regarding the distribution of task RT differences (i.e., the rule-based efficiency effect). We tested this by assessing the homogeneity of variances using pair-wise Brown-Forsythe tests. The comparisons demonstrated that the variance of rule-based efficiency effects in the young age group was significantly different from that in the old age group, *F* (1, 46) = 9.06, *p*_*bf*_ = 0.013. The variance of rule efficiency in the middle age group was neither significantly different from the young age group, *F* (1, 46) = 1.17, *p*_*bf*_ = 0.86 nor from the old age group, *F* (1, 46) = 3.53, *p*_*bf*_ = 0.20.

### 3.3. DDM

Descriptive and interference statistics for DDM parameters per task are depicted in Table 1.

#### 3.3.1. Boundary separation simulation data

**FIGURE 4:**
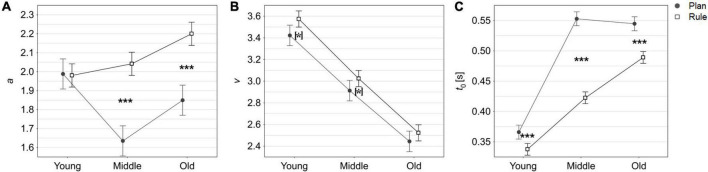
Drift diffusion model (DDM) parameter estimates from simulated datasets. Error bars represent 95% confidence intervals. [*] denotes uncorrected *p*-values < 0.05; *** denotes Bonferroni corrected *p*-values < 0.001 of within-group *t*-test task comparisons. **(A)** DDM boundary separation parameter (*a*) by age group and task. **(B)** DDM drift rate parameter (*v*) by age group and task. **(C)** DDM non-decision time parameter (*t*_0_) in seconds by age group and task. Drift rate, *v*: the rate of information accumulation (speed of the decision process), boundary separation, *a*: the distance between decision thresholds (response caution), and the duration of non-decision components *t*_0_: stimulus encoding, preparation of motor response, visualization (action encoding).

With the exception of the young age group, boundary separation based on simulated rule-task RT data was significantly higher than those based on simulated plan-task data (see Figure 4A).

#### 3.3.2. Drift rate simulation data

The difference in mean drift rates between tasks (0.15 in the young age group, 0.11 in middle age group and 0.08 in the old age group) appears to decrease with age. In the young and middle age groups, drift rates in the rule-task were higher than in the plan-task but the effect was not significant after Bonferroni correction for multiple comparisons.

In the old age group, the difference in drift rates between the plan-task and the rule-task was not significant (see Figure 4B).

#### 3.3.3. Non-decision time simulation data

In all age groups, non-decision times based on simulated rule-task RT data were significantly lower than those based on simulated plan-task data (see Figure 4C).

## 4. Discussion

In line with our initial studies, we found a significant advantage of rule- over plan-based action selection in young and middle-aged participants. Our DDM and RT results suggest altered motor cognitive mechanisms in older adults. As a function of age, we found an increased divergence between plan and rule RTs. But the observed divergence does not support our hypothesis of a general advantage of the rule-based approach. On the contrary, the quite robust rule-based efficiency effect vanished in the old age group. Instead, in older age groups, differences between the two approaches to action selection went in both directions: for some participants, rule-based action selection was initiated faster, while for others, plan-based action selection seemed more efficient. While these results affirm that explicit rules have the potential to facilitate response initiation in both young and older adults, this is apparently not a trait common to the whole sample of older adults. Thus, lifespan factors that may alter the effect of rule-based efficiency for action selection should be specified.

A recent meta-analysis (Theisen et al., 2021) on age differences in DDM parameters showed a general increase in both response caution (*a*) and non-decision parameters but more complex patterns of age differences for drift rates: Older adults tended to favor accurate over fast responses and took longer in terms of non-decisional components, i.e., movement encoding. Interestingly, modulatory effects of task were found for the speed of information uptake (*v*). Compared to younger adults, performance by older adults was decreased in perceptual or memory tasks but superior in lexical decision tasks.

Similarly, in our study, age-specific differences between tasks could be observed on a descriptive level for the speed of information processing (*v*) with differences between tasks being reduced in the old age group. One may speculate that perhaps for the pre-movement phase (the period of time in which the decision process takes place), the rule-task places greater loads on memory functions (retrieval and maintenance of the if-then rule, working-memory) thereby increasing task difficulty and contributing to the loss of rule-efficiency effect with age. Thus, altered speed of information uptake and processing could be one critical factor determining the age-related change of rule-based action selection efficiency.

Instead, non-decision components (*t*_0_) were faster in the rule-task than in the plan-task across all age groups, suggesting that rule-based action selection generally has lower demands in the pre-movement phase. Perhaps this could be due to early movement parameters being prescribed by the rule, which may facilitate movement encoding (if…, then put the thumb on the same side…vs. choose the most comfortable way to…). Our results showing a higher non-decision time in older adults are consistent with the behavioral findings of Frolov et al. (2020), who demonstrated age-related slowing of motor initiation prior to hand movements. Frolov et al. (2020) also showed that these behavioral changes were accompanied by increased motor brain response time in mu and theta bands before movement initiation.

Furthermore, all age groups but the youngest demonstrated higher response caution (*a*) for rule-based than for plan-based action selection. On a descriptive level, the finding of an increase in response caution coinciding with the oldest group is also in line with previous research on risk perception in older adults e.g., (Bonem et al., 2015) and has been reported for some other motor cognitive decision tasks as well e.g., (Finkel et al., 2019). It could be speculated that a relatively larger increase in response caution in the rule-task as a function of older age presents one additional factor determining the age-related change of rule-based action selection efficiency.

In line with our findings of more general changes in behavioral parameters across groups, it is known that motor performance decreases from young adulthood to old age mirroring results from cognitive research (Leversen et al., 2012). Findings by Ren et al. (2013) suggest that older adults require more cognitive resources than younger adults to carry out the same motor tasks. Older age goes along with a brain volume loss (Hedman et al., 2012), and it has been discussed that older adults may adopt more adaptive strategies to better compensate for age-related changes in motor planning (Wang et al., 2020). At the same time, our task specific data suggests a more individualized preference for one of the two strategies to action selection increasing with age. The theoretical framework building upon so called Neural Darwinism could be one way to interpret such results. The individualization and specialization in older age could be viewed as a process of selection taking place inside the nervous system. Perhaps, the key areas of structural decline and nature of compensatory brain mechanisms may determine which approach to action selection (rule versus plan-based) remains or becomes more successful in older age. One related mechanism playing a potential role in this could be a pronounced use of one or the other strategy shaped by the type of engagement during daily life (for a similar argument as the described see Leversen et al., 2012).

Although in parts unexpected, most of our results can be embedded in the more general literature. However, the current results and task-specific patterns of parameters across the lifespan must, of course, be replicated. Interestingly, most older adults seemed to prefer one approach over the other, without a specific direction observable over the whole sample. Since there is no general efficiency advantage for one task (i.e., rule-based) in the healthy older age group, determining whether a person with difficulties in action selection is using the optimal approach will require thorough individualized testing. Similarly, identifying the more efficient approach for individual patients, would enable the selection of the more efficient approach as a compensatory action selection route. For example, when older patients struggle to adapt to new aids, like walkers, they could be supported by short and easy rules (e.g., “If I am walking down a steeper hill, then I will apply the walker’s brake”). However, to prevent confusion it is inevitable to assess whether the patient can actually benefit from rules when it comes to action selection. These ideas remain to be tested with larger sample sizes and a greater number of trials per participant to enable straight forward and robust DDM parameter estimation. Critically, these conditions greatly complicate the implementation of this experimental setting in clinical contexts that typically coincide with small sample sizes and a reduced number of trials due to diminished participant endurance. Furthermore, large group studies do not solve the problem of masked efficiency effects on the group level, particularly in older age groups due to the observed increase in divergence between plan and rule-based efficiency effects.

## 5. Conclusion

Initially, we hypothesized an increase in rule-based action selection efficiency relative to plan-based action selection efficiency, coinciding with increasing age.

Efficiency advantages in terms of shorter RTs diverged in the older age group: while for some older participants, rule-based action selection was initiated faster, others were quicker in plan-based action selection. Perhaps, the age-related changes we have described using the DDM in the context of the RPMC paradigm are on a more general level of cognitive functions (e.g., memory) that contribute differently to the specific motor cognitive task at hand (e.g., rule versus plan). These ideas need further investigation.

## Data availability statement

The raw data supporting the conclusions of this article are available from https://doi.org/10.5281/zenodo.7611706.

## Ethics statement

The studies involving human participants were reviewed and approved by University of Konstanz Ethics Committee. The patients/participants provided their written informed consent to participate in this study.

## Author contributions

JS: study design, data acquisition, analysis, and interpretation and writing. SS: analysis and writing. JR: study idea, study design, and interpretation and writing. All authors contributed to the article and approved the submitted version.

## Footnotes

1
http://www.translucent.ca

2
https://www.frontiersin.org/articles/10.3389/fpsyg.2018.00309/full
