# Supplementary material for: Does aging amplify the rule-based efficiency effect in action selection?
Source: Front Psychol. 2023 Mar 1;14:1012586. doi: 10.3389/fpsyg.2023.1012586 (PMC10014753; doi:10.3389/fpsyg.2023.1012586)
Supplement: Supplementary file 1 [file Data_Sheet_1.pdf]

Supplementary Material:

Does Aging Amplify the Rule-based Efficiency Effect in Action Selection?

1 Overview

This supplement provides additional data on the pre-processing procedure (trial exclusion), participant condition assignment, grip error rates, hand and task-sequence effects, and details on the simulation procedure used for DDM analysis.

2 Data Pre-Processing Procedure

2.1 Reaction time analysis

The following pre-processing steps were performed in the following sequence for reaction time (RT) analysis:

First, trials containing technical errors (e.g., shutter goggles partially opening or remaining closed; light failures on the rotation apparatus) were excluded. Second, "other" errors (e.g., trials affected by environmental confounds such as loud noises; trials in which participants were otherwise distracted e.g., scratched themselves or adjusted the shutter goggles; trials in which participants released the response pad button early i.e., before the goggles opened). Third, all trials with incorrect grips (i.e., trials in which participants performed an overhand grip when an underhand grip would have led to a comfortable post-handle-rotation end-state) were excluded. Finally, each participant's data was tested for outliers in each task*grip combination with the Extreme Studentized Deviate test (ESD; Rosner, 1983). The identified outliers were removed prior to data set aggregation. See **Supplementary Figure 1 and Supplementary Table 1** for further details.

2.2 DDM analysis

Different from the pre-processing steps implemented for reaction time analysis (described above), we did not remove trials with grip errors, as we aimed to model correct and incorrect responses. Rather we implemented the same outlier removal procedure that we used for correct trials (i.e., ESD based outlier detection).

To recap, we first removed trials containing technical errors. Second, we removed "other" errors. Third, each participant's data was tested for outliers with the ESD test in each task*grip combination. This was done separately for trials with incorrect and correct grips.

We then combined the pre-processed data sets of respective participants in each age group into a young, middle, and old super subject dataset. As outliers can strongly bias DDM analysis results, we performed a separate outlier removal step on each super subject data set using boxplots to identify the outliers. Following the recommendations outlined in Voss et al. (2015), we identified outlier trials from their log-transformed values to identify fast outliers with greater sensitivity.

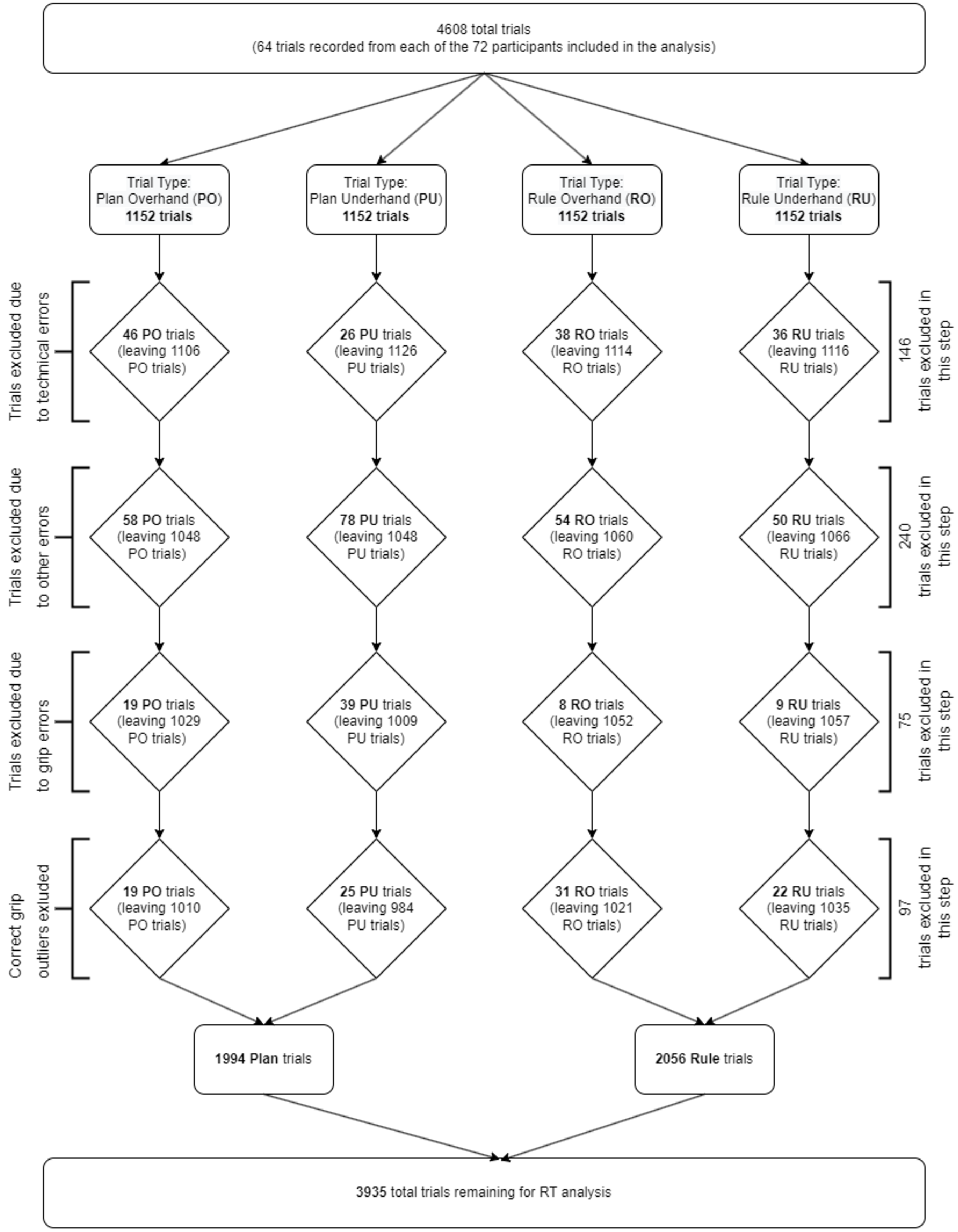

Supplementary Figure 1 The figure depicts the sequence of pre-processing steps and shows the number of trials excluded in each step. Note that outliers were determined on a per-participant basis

and identified using the Extreme Studentized Deviate test (Rosner, 1983), separately for each task*grip combination. Not shown in this figure: two incorrect grip outliers (both plan underhand; one in the young age group and one in the old age group).

Supplementary Table 1.

Number of excluded trials per age group task and grip

	Task	Grip	**Young**	**Middle**	**Old**	Row totals
Grip Errors	Plan	Overhand	6	6	7	19
		Underhand	11	10	18	39
	Rule	Overhand	2	3	3	8
		Underhand	5	0	4	9
Total Grip Errors			24	19	32	75
Correct Grip Outliers	Plan	Overhand	4	7	8	19
		Underhand	6	12	7	25
	Rule	Overhand	9	14	8	31
		Underhand	6	8	8	22
Total Correct Grip Outliers			25	41	31	97
Incorrect Grip Outliers*	Plan	Overhand	0	0	0	0
		Underhand	1	0	1	2
	Rule	Overhand	0	0	0	0
		Underhand	0	0	0	0
Total Incorrect Grip Outliers			1	0	1	2
Technical Errors	Plan	Overhand	16	9	21	46
		Underhand	13	8	5	26
	Rule	Overhand	18	10	10	38
		Underhand	13	6	17	36
Total Technical Errors			60	33	53	146
Other Errors	Plan	Overhand	18	17	23	58
		Underhand	28	26	24	78
	Rule	Overhand	20	14	20	54
		Underhand	8	23	19	50
Total Other Errors			74	80	86	240
Column totals		Column totals	184	173	203	560

Note. The table shows descriptive statistics of trial exclusion for RT Analysis. The reason for exclusion is given in the far left column.
*Incorrect grip outliers are included for completeness and relevance for the DDM data set.

Supplementary Table 2.

DDM Super subject level outlier removal

Response	Task	Grip	Age Group			Row totals
			**Young**	**Middle**	**Old**	
Correct	Plan	Overhand	17	1	1	19
		Underhand	13	3	4	20
	Rule	Overhand	8	8	5	21
		Underhand	5	6	7	18
Incorrect	Plan	Overhand	2	0	1	3
		Underhand	1	2	0	3
	Rule	Overhand	0	0	0	0
		Underhand	1	0	0	1
Total			47	20	18	85

3 Participant Condition Assignment

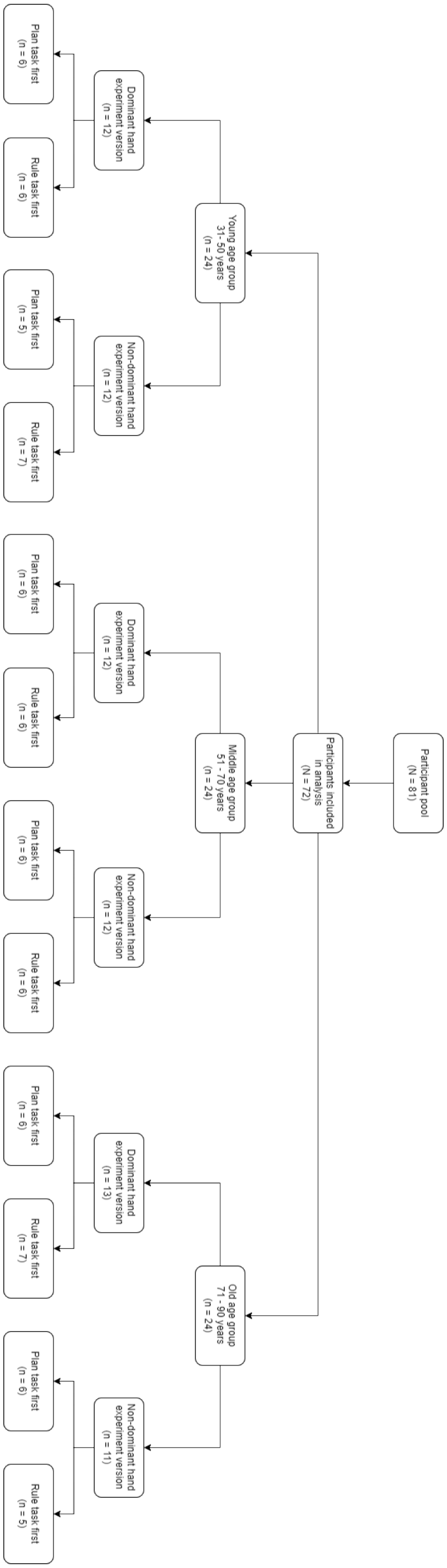

Supplementary Figure 2 The figure shows the assignment of participants in each age group to the different experiment versions.

4 Grip Error Rates

Supplementary Table 3.

Grip error rates by task and age group

	Task	Young	Middle	Old	Mean Task Error Rates (%)
Grip Error Rates (%)	Plan	2.45	2.26	3.60	2.77
	Rule	0.99	0.42	1.00	0.80
Mean Group Error Rates (%)		1.72	1.72	1.34	

Supplementary Table 4.

Grip error rates by task age group and grip

	Task	Grip	Young	Middle	Old	Mean Task Error Rates (%)
Grip Error Rates (%)	Plan	Overhand	1.71	1.68	2.06	1.82
		Underhand	3.21	2.86	5.07	3.71
	Rule	Overhand	0.58	0.83	0.85	0.75
		Underhand	1.38	0.00	1.15	0.84
Mean Group Error Rates (%)			1.72	1.34	2.28	

5 Hand and Task-Sequence Effects

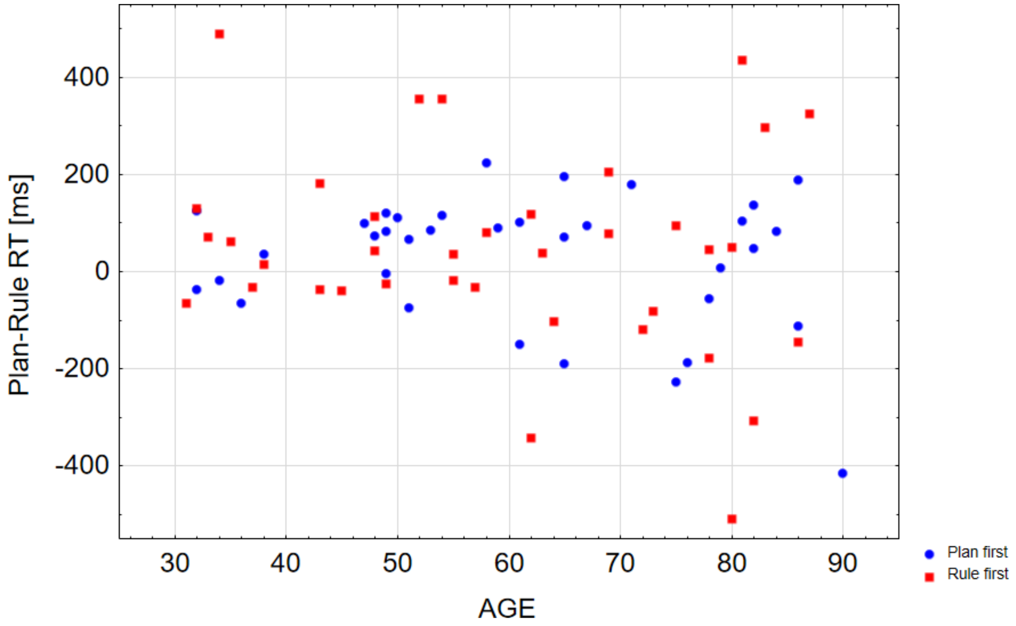

Supplementary Figure 3 There was no significant effect of task sequence (plan task first or rule task first) on the difference between plan and rule RTs (i.e., the rule efficiency effect calculated from rule RTs subtracted from plan RTs for each subject). All age groups: U = 629, *p* = 0.840; young: U = 68.00, *p* = .862; middle: U = 69.00, *p* = .885; old: U = 71.00, *p* = .977

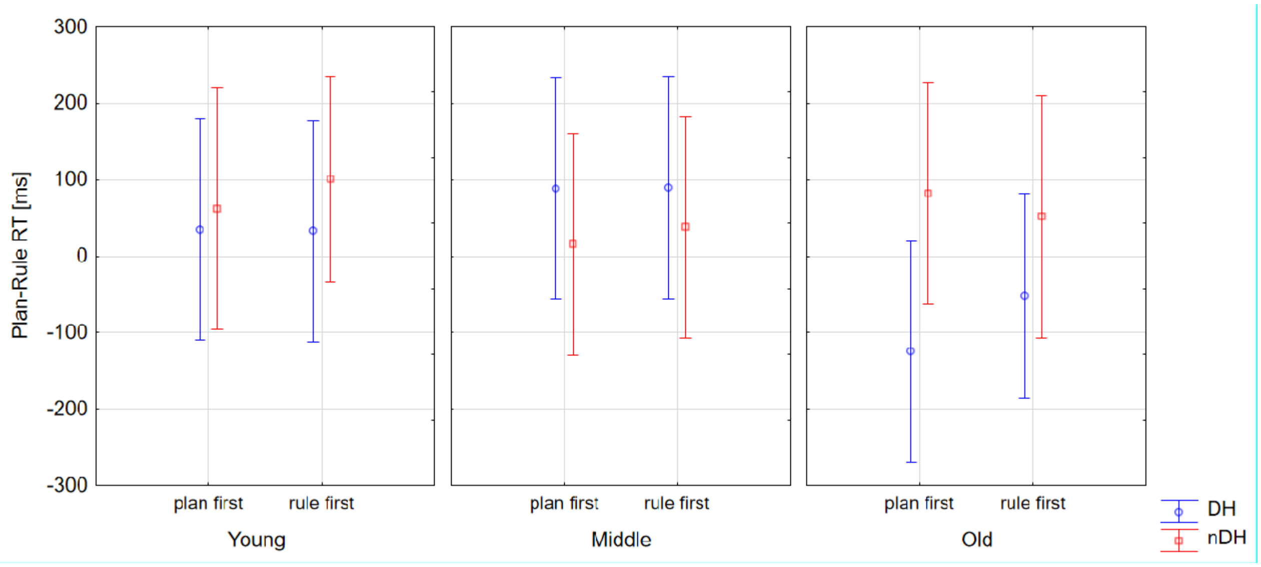

Supplementary Figure 4 There were no significant differences in rule efficiency (e.g., the difference between plan and rule RTs calculated from rule RTs subtracted from plan RTs for each subject). Young dominant hand (DH): U = 18, *p* = .936; young non-dominant hand (nDH): U = 15, *p* = .927; middle DH: U = 14, *p* = .575; middle nDH: U = 17, *p* = .936; old DH: U = 16, *p* = .520; old nDH: U = 15, *p* = .927.

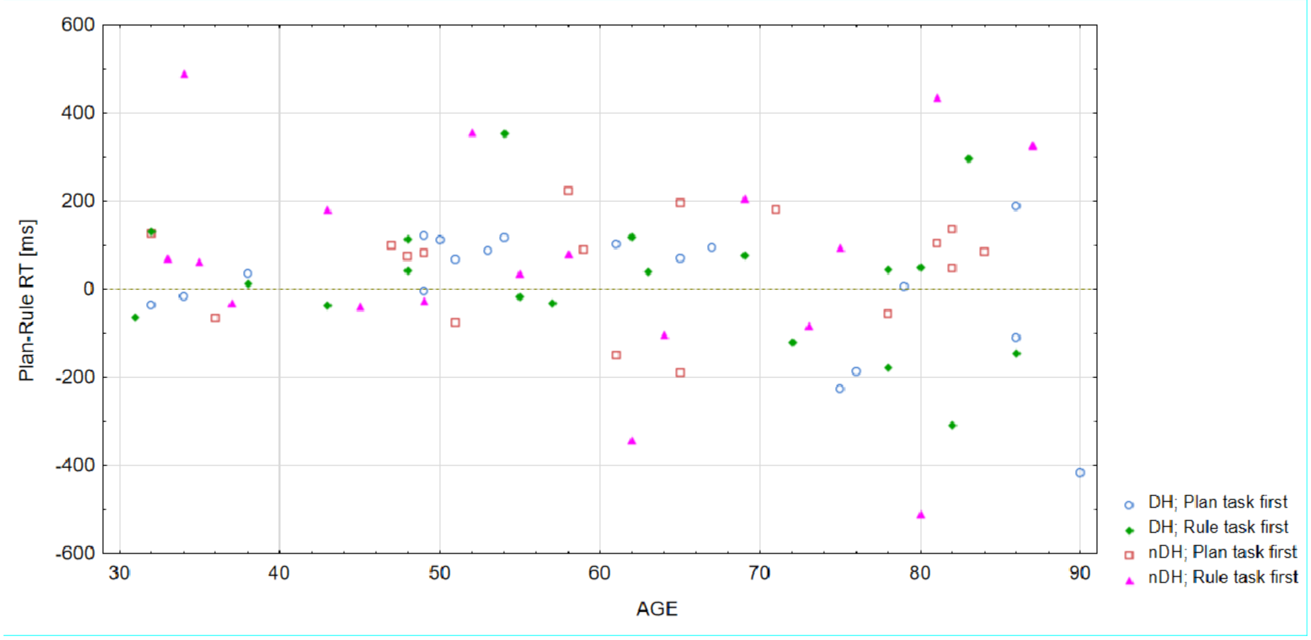

Supplementary Figure 5 Rule efficiency advantage for hand by sequence

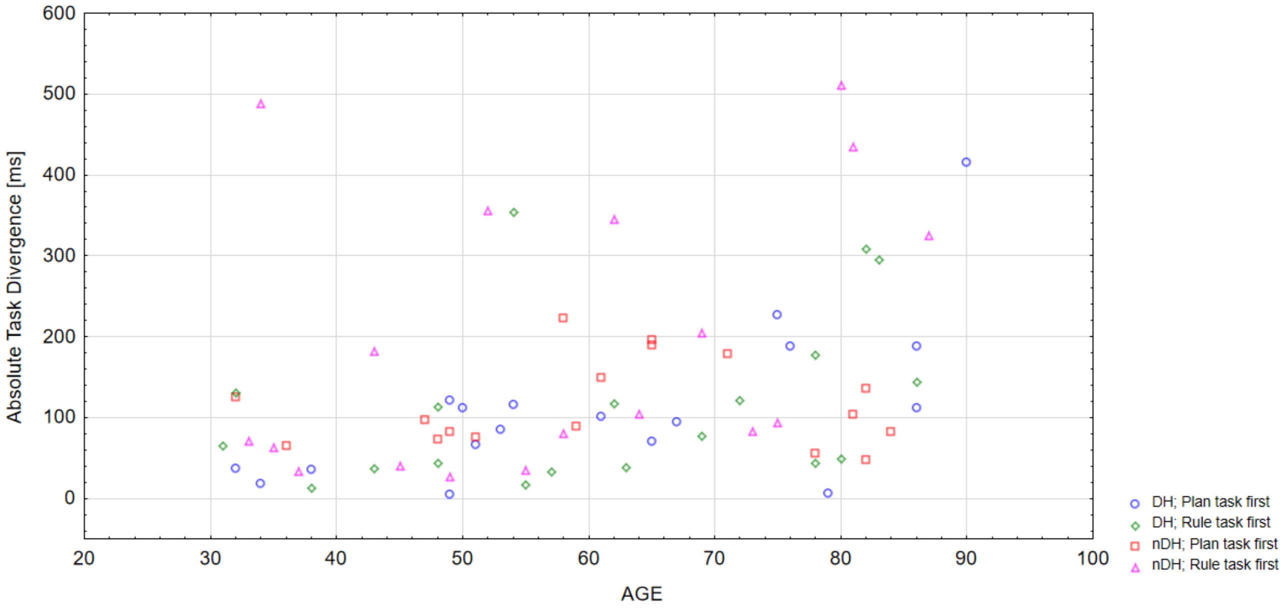

Supplementary Figure 6 Task divergence for hand by sequence

6 Simulation Details

In the following, we supply the commands passed to the construct-samples tool (available from https://www.psychologie.uni-heidelberg.de/ae/meth/fast-dm/index.html) to create the simulated data sets. The parameter values used in the commands were calculated from young, middle, and old super subject data sets using *fast-dm-30.2*. For a full overview of function parameters visit https://www.psychologie.uni-heidelberg.de/ae/meth/fast-dm/index.html#cs.

The data and additional notes on replicating the DDM analysis including the simulation are available from https://doi.org/10.5281/zenodo.7611706.

6.1 Young age group

6.1.1 Rule task

construct-samples.exe -a 1.798371 -z 0.5 -v 3.485587 -t 0.355786 -d -0.004386 -Z 0 -V 0.918757 -T 0.276720 -r -n 4096 -N 24 -o "%d_young-rule-samples4096_bf-rr.txt"

6.1.2 Plan task

construct-samples.exe -a 1.767115 -z 0.5 -v 3.282453 -t 0.409138 -d 0.040028 -Z 0 -V 0.850918 -T 0.333761 -r -n 4096 -N 24 -o "%d_young-plan-samples4096_bf-rr.txt"

6.2 Middle age group

6.2.1 Rule task

construct-samples.exe -a 1.9122 -z 0.5 -v 2.9131 -t 0.4222 -d -0.0251 -Z 0 -V 0.411 -T 0.4045 -r -n 4096 -N 24 -o "%d_middle-rule-samples4096_bf.txt"

6.2.2 Plan task

construct-samples.exe -a 1.5477 -z 0.5 -v 2.7901 -t 0.5697 -d 0.0222 -Z 0 -V 0.0324 -T 0.4691 -r -n 4096 -N 24 -o "%d_middle-plan-samples4096_bf.txt"

6.3 Old age group

6.3.1 Rule task

construct-samples.exe -a 2.0152 -z 0.5 -v 2.4184 -t 0.4906 -d -0.0434 -Z 0 -V 0.3106 -T 0.5036 -r -n 4096 -N 24 -o "%d_old-rule-samples4096_bf.txt"

6.3.2 Plan task

construct-samples.exe -a 1.7359 -z 0.5 -v 2.292 -t 0.5954 -d 0.0786 -Z 0 -V 0.1867 -T 0.6288 -r -n 4096 -N 24 -o "%d_old-plan-samples4096_bf.txt"
